# Time estimation in mild Alzheimer's disease patients

**DOI:** 10.1186/1744-9081-5-32

**Published:** 2009-07-28

**Authors:** Luana Caselli, Luca Iaboli, Paolo Nichelli

**Affiliations:** 1 Department of Biomedical Sciences and Advanced Therapies, University of Ferrara, Via Fossato di Mortara, Ferrara, Italy; 2 Department of Emergency Medicine, Arcispedale Santa Maria Nuova, Via Risorgimento, Reggio Emilia, Italy; 3 Department of Neurosciences, University of Modena and Reggio Emilia, Nuovo Ospedale Civile Sant'Agostino-Estense Via Giardini, Modena, Italy

## Abstract

**Background:**

Time information processing relies on memory, which greatly supports the operations of hypothetical internal timekeepers. Scalar Expectancy Theory (SET) postulates the existence of a memory component that is functionally separated from an internal clock and other processing stages. SET has devised several experimental procedures to map these cognitive stages onto cerebral regions and neurotransmitter systems. One of these, the time bisection procedure, has provided support for a dissociation between the clock stage, controlled by dopaminergic systems, and the memory stage, mainly supported by cholinergic neuronal networks. This study aimed at linking the specific memory processes predicted by SET to brain mechanisms, by submitting time bisection tasks to patients with probable Alzheimer's disease (AD), that are known to present substantial degeneration of the fronto-temporal regions underpinning memory.

**Methods:**

Twelve mild AD patients were required to make temporal judgments about intervals either ranging from 100 to 600 ms (short time bisection task) or from 1000 to 3000 ms (long time bisection task). Their performance was compared with that of a group of aged-matched control participants and a group of young control subjects.

**Results:**

Long time bisection scores of AD patients were not significantly different from those of the two control groups. In contrast, AD patients showed increased variability (as indexed by increased *WR *values) in timing millisecond durations and a generalized inconsistency of responses over the same interval in both the short and long bisection tasks. A similar, though milder, decreased millisecond interval sensitivity was found for elderly subjects.

**Conclusion:**

The present results, that are consistent with those of previous timing studies in AD, are interpreted within the SET framework as not selectively dependent on working or reference memory disruptions but as possibly due to distortions in different components of the internal clock model. Moreover, the similarity between the timing patterns of elderly and AD participants raises the important issue of whether AD may be considered as part of the normal aging process, rather than a proper disease.

## Background

Time information processing implies the interaction among diverse cognitive systems including memory, which greatly supports the operations of hypothetical internal timekeepers.

Memory is associated with both medio-temporal and frontal regions. Studies in humans have revealed that these brain regions play an important role in temporal information processing, so that it has been suggested that working memory and time perception depend on the same neural networks [1]. For example, two well-known amnesic patients, H.M., who became severely amnesic after bilateral removal of the medial temporal cortex [2] and B.W., who started suffering amnesia after the removal of a cyst in the third ventricle [3], showed severe time underestimation.

Research on the brain structures involved in temporal information processing has led to the formulation of a modular information-processing model, based on the Scalar Expectancy Theory (SET, [4]), postulating the existence of a memory component that is functionally separated from other processing stages.

**Figure 1:**
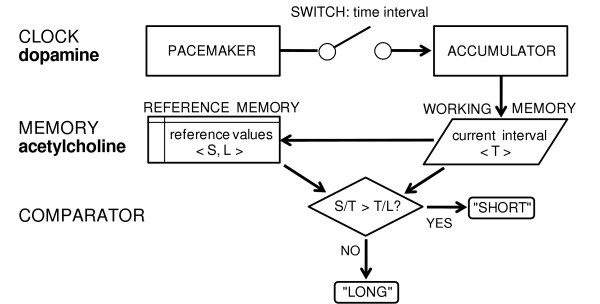
**SET information-processing model applied to time bisection tasks**. In the SET model, a pacemaker emits pulses that are gated by a switch during the current to-be-timed interval (T) and sent to an accumulator. The corresponding number of pulses are stored in working memory and compared with that of reference short (S) and long (L) intervals previously stored in reference memory during the training phase. According to a similarity rule based on the S/T and T/L ratios, T will be estimated to be more similar to either of the two reference values and a response (either "SHORT" or "LONG") will be produced. According to STT model, the clock stage is controlled by the dopaminergic system, whereas the memory stage is dependent on the cholinergic system.

According to this model, an internal clock stage includes a pacemaker that emits pulses in time and an accumulator that receives the pulses gated by a switch during a certain time interval. The memory stage is formed by a working memory component retaining the accumulator pulse count for the current timing operations and by a reference memory system storing distributions of previous clock readings for comparison (figure 1).

The search for the neuronal underpinnings of temporal information processing has been producing a growing body of research, mainly concerning the physiological correlates of the internal clock and memory both in animals (e.g., [5-11]) and humans (e.g., [12-24]).

SET has devised several experimental procedures to map the different cognitive stages involved in time encoding onto cerebral regions and neurotransmitter systems. One of these, the time bisection procedure, applied to pharmacological tests in animals, has provided support for a dissociation between the clock stage, affected by dopaminergic manipulations, and the memory stage, mainly influenced by cholinergic manipulations [5,7,8]. In a time bisection task, subjects are required to estimate specific durations as more similar to either of two short (S) and long (L) reference intervals. SET assumes that, to solve the task, two (S and L) reference interval distributions are stored in memory. For each current to-be-estimated duration (T), the pulse count in working memory is compared (through a comparator stage) against two samples, one extracted from the memory store for short durations (S/T) and the other from the memory store for long durations (T/L). Estimation responses are given according to the greatest time ratio produced (see figure 1, for SET applied to time bisection tasks). Plotting the proportion of *long *responses yields a psychometric function that is typically sigmoidal in shape and from which significant time estimation parameters can be extracted.

SET states that the memory stage is fundamental for correctly processing time and the bisection task was originally designed to assess this, based on the assumption that the comparison of intermediate durations with the short and long anchors maximizes reliance on memory for reference durations [25]. Pharmacological studies in rats investigating memory mechanisms in time bisection, showed that decreasing cholinergic activity by administration of atropine caused overestimation and increased variability in temporal judgments (i.e., bisection functions respectively shifted rightward and decreased in slope), whereas increasing cholinergic activity by physostigmine led to time underestimation and decreased variability [5,26,27]. Congruently, in a study employing the peak-interval experimental procedure [11], in which the animal's response rate typically increases as a function of the expected time of reinforcement (peak rate time), atropine administration increased the variability of temporal discrimination and shifted peak times rightward in a dose-dependent fashion. Physostigmine administration produced opposite effects. Moreover, rats with lesions to the nucleus basalis magnocellularis (NBM) as well as those with lesions to the frontal cortex (FC) were shown to overestimate temporal intervals (i.e., rightward shift of the estimated time of reinforcement). In contrast, hippocampal lesions in the medial septal area and fimbria-fornix (MSA, FFx) decreased peak times (i.e., psychometric functions shifted leftward) and produced a total inability to regulate the trial-by-trial criterion for temporal estimates [10,22]. These findings, which have been replicated [28,29], suggest that frontal and hippocampal systems are both involved in the SET memory stage, but in complementary ways. The frontal system seems to be directly responsible for storing temporal information in the reference memory component. Cholinergic activity in the frontal cortex, as measured by sodium-dependent high-affinity choline uptake, has been shown to be proportional to storage errors in the content of temporal reference memory [9]. In contrast, the hippocampal system plays a major role in the timing operations of the working memory component, by recording temporal information relevant to a single event. In the peak-interval with a gap procedure, in which trials with a duration retention period are additionally introduced to study working memory temporal functions, hippocampal lesions caused amnesia for the interval prior to the gap [10].

NBM and MSA are the principal areas of projection (to the frontal cortex and to the hippocampus, respectively) of the basal forebrain cholinergic system (BFCS), a region that has been receiving considerable attention because of its substantial degeneration in Alzheimer's disease (AD) patients [30].

Disruption of temporal processing has been observed in AD [31-37]. For example, patients with a diagnosis of mild-to-moderate probable AD showed increased variability and impaired accuracy in timing tasks [34-37]. However, most of the studies investigating time estimation in AD have employed duration stimuli in the order of several seconds, that typically require increased attentional and mnemonic demands with respect to shorter intervals [38-40], making it difficult to specifically mark out deficits of temporal perception while minimizing generalized impairments of higher cognitive functions. Moreover, despite that a substantial body of research has been dedicated to investigate timing abilities in AD patients, there is very scant evidence for their performance in experimental procedures specifically developed in the SET framework. In one study, bisection tasks were used to assess time discrimination for both short (millisecond range) and long (second range) intervals in one patient with probable frontotemporal dementia [41]. However, likely due to the single-case design and the temporal stimuli employed in the study, no significant results were found.

Keeping in mind that the nature and procedures employed in animal pharmacological studies are substantially different from those of human neuropsychological studies, testing time estimation in AD patients might be helpful to shed some light on the role of the memory components postulated by SET.

In the present research, we investigated time estimation in a group of patients with probable AD by using two (sub- and supra-second) time bisection tasks and compared their performance with that of both aged-matched and young control subjects. The employment of both millisecond and second durations allowed to better isolate temporal perception mechanisms from more generalized attentional and mnemonic processes.

Results were interpreted within the theoretical framework of SET.

## Methods

### Subjects

We tested 12 patients (four males and eight females, mean age 70.6 years-range 60–78 years-; mean educational level 5.7 years) with a clinical diagnosis of probable mild Alzheimer's Disease and compared their performance with that of 12 normal participants matched for age (mean 71.4 years; range 60–81), educational level (mean 5 years; range 3–7 years) and gender (five males and seven females). A control group of 12 young participants (mean age 28.4 years; range 24–35 years) was additionally examined.

Before taking part to the current experiment, all patients had received a standardized neurological examination, a computed tomography (CT) or magnetic resonance imaging (MRI) scan and a standardized neuropsychological evaluation including paired-associate learning, visual denomination, line orientation judgment and dual task tests. General cognitive functioning and severity of dementia had been assessed through Mini-Mental State Examination [42], Milan Overall Dementia Assessment [43] and Activity Daily Living [44]. The diagnosis of probable mild Alzheimer's Disease was based on the clinical criteria of the Diagnostic and Statistical Manual of Mental Disorders developed by the American Psychiatric Association (DSM IV-TR, APA, 2000). Table 1 summarizes the scores of the patients on these tests and scales.

**Table 1:** Neuropsychological test scores of AD patients

	**MMSE**	**MODA**	**ADL**	**VD**	**PAL**	**LOJ**	**DT**
AVERAGE	21.11	83.09	7.25	15.5	7.83	18.08	85.82
S.D.	1.25	6.07	1.23	3.20	2.00	6.80	15.89
RANGE	19.47 23.27	73.9 97.4	5.5 10	9 20	5.5 11.5	5 30	54.8 107.5

**MMSE **= Mini Mental State Examination; **MODA **= Milan Overall Dementia Assessment; **ADL **= Activities of Daily Living; **VD **= Visual Denomination; **PAL **= Paired Associate Learning; **LOJ **= Line Orientation Judgement; **DT **= Dual Task.

The study was approved by the local ethical committee.

### Apparatus

Tasks were designed and implemented on a Power Macintosh computer by using Superlab software (Cedrus Corporation, 1992).

The participants were asked to sit in front of a 21-inches computer screen at a distance of about 50 cm and were administered two time bisection tasks, requiring to classify different time durations as more similar to a short or a long reference interval:

#### 1. Short time bisection task

Reference short and long intervals were 100 and 600 ms, respectively. Time durations to be discriminated were 9, ranging from 100 to 600 ms and set at 62.5 ms increments.

#### 2. Long time bisection task

Reference short and long intervals were 1000 and 3000 ms, respectively. Time durations to be discriminated were 9, going from 1000 to 3000 ms and set at 250 ms increments.

### Bisection procedure

The onset and the offset of intervals were marked by a 10-ms 900-Hz tone and a 1 × 1 cm white square appeared at the centre of the computer screen for the duration of each presented interval. The response "SHORT" or "LONG" was given verbally.

A training phase preceded the experiment, during which the participants were exposed to five presentations of both the reference short and long stimuli, followed by a twenty-trial practice session in which the participants were required to classify as short or long these reference intervals. All participants correctly classified each presented interval.

After the training, ten blocks of experimental trials were administered, during which all 9 intervals were randomly presented. Thus, both the short and the long time bisection tasks consisted of 90 discrimination trials. The two reference intervals were presented before the beginning of each trial block to make the subjects re-adjust their temporal judgment criterion.

### Bisection scoring

The number of times each participant classified an interval as long was plotted against interval duration. The proportion of long responses P(L) was fit with a sigmoidal psychometric function (P(L) = e^(β*D)^/1-e^(β*D)^) using an iterative least-square method applied to an unbiased logistic regression for time duration. Then, based on each individual function, the following time bisection parameters were computed:

*1) Bisection point (BP)*, corresponding to the interval classified as "long" on 50% of trials, (the value on the abscissa relating to P(L) = 0.5).

*2) Difference limen (DL)*, that is half the difference between the intervals classified as "long" on 75% and on 25% of trials, respectively. *DL *measures the subjective ability to discriminate between different time durations. The higher it is, the worse the ability to distinguish one interval from the others.

*3) Weber ratio (WR)*, obtained by dividing the *DL *by the *BP*. Since *DL *is expected to vary as a function of *BP *(as according to Weber's Law), *WR *represents temporal sensibility normalized on the specific estimated intervals, thus allowing for direct comparison of timing variability across various anchor pairs.

*4) Precision*, that is the consistency of responses over successive trials, represented by the distribution of points around the curve. It is defined as the percentage of the total variance explained by the logistic model (1-SSE/SST × 100, where SSE is the sum of squares of the error and SST is the total sum of squares).

## Results

**Figure 2:**
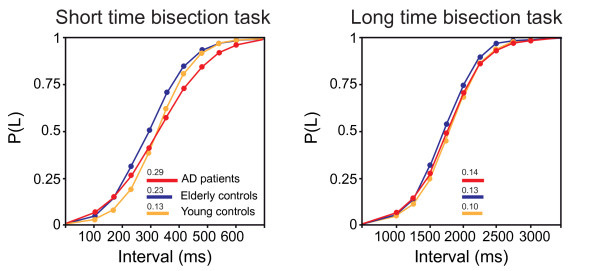
**Time bisection psychometric functions**. Typical S-shaped psychometric functions obtained for the three groups in the short and long time bisection tasks. Time intervals to be discriminated are plotted against the proportion of long responses P(L). Length of horizontal bars and numbers on top respectively indicate mean *Difference Limen *and *Weber Ratio *values for each group.

**Figure 3:**
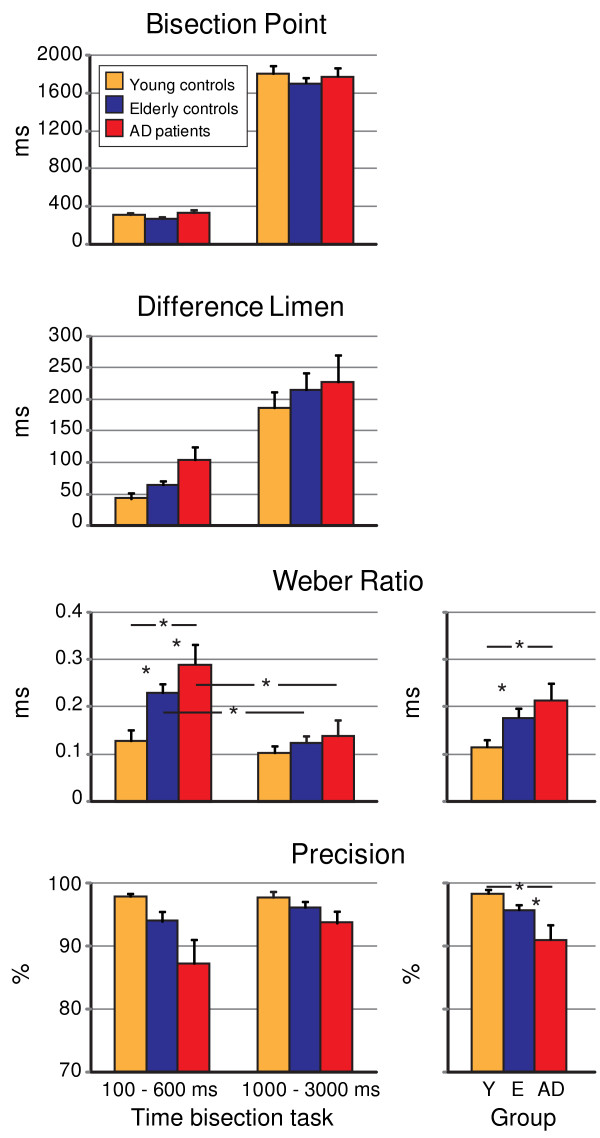
**Time bisection scores**. Bisection Point, Difference Limen, Weber Ratio and Precision scores (mean ± s.e.m.) of Young controls, Elderly controls and AD patients in the short and long time bisection tasks. Asterisks indicate significant between- or within-subject differences (5% alpha level), as revealed by the two-way ANOVAs. *Weber Ratio *and *Precision group main effects *are also shown.

Psychometric functions resulting from the responses given by two AD patients in both bisection tasks were not typically S-shaped as those produced by all other subjects, including patients and controls. As a consequence, their bisection scores were discarded from the AD patients' sample. Statistical analyses were thus performed on data obtained from 10 AD patients, 12 elderly controls and 12 young controls. For each selected bisection parameter (*BP*, *DL*, *WR *and *Precision*), a two-way ANalysis Of VAriance (ANOVA), with *group *(*young *controls, *elderly *controls and *AD patients*) as between-subject factor and *task *(*short *and *long *time bisection) as within-subject factor, was run to compare the average scores of the three groups in the two time bisection tasks. Post-hoc contrasts were performed by means of Fisher's Protected Least Square Difference (PLSD) test. Psychometric functions obtained for each group in the two time bisection tasks are illustrated in figure 2 and main statistical results are shown in figure 3.

Average group scores reported below are always followed by ± standard error values.

### Bisection point

No significant difference in interval evaluation accuracy, as indicated by this parameter, was found among the 3 groups in either time bisection task (*GROUP main effect: F(2, 31) = 0.8, p = 0.4; GROUP × TASK interaction: F(2, 31) = 0.2, p = 0.8*; Figure 3).

### Difference limen

Despite progressive decreasing in the ability to discriminate intervals of elderly participants and AD patients with respect to young controls, as indicated by their respective *DL *values, no significant *DL *difference was found among the 3 groups in either time bisection task (*GROUP main effect: F(2, 31) = 2.1, p = 0.1; GROUP × TASK interaction: F(2, 31) = 0.2, p = 0.8*).

However, *DL *for long time estimates (209 ± 18 ms) was, on average, significantly higher than for short time estimates (70 ± 8 ms) (*TASK main effect: F(1, 31) = 52.4, p = 0.00*).

### Weber ratio

Analysis of *WR *indicated a significant main effect of *GROUP *(*F(2, 31) = 8.2, p = 0.001*): overall, young controls showed increased timing sensitivity (0.12 ± 0.01 ms) with respect to both elderly controls (0.18 ± 0.02 ms) and AD patients (0.21 ± 0.03 ms). In addition, a significant *GROUP × TASK interaction *(*F(2, 31) = 3.6, p = 0.04*) revealed that *WR *differences among groups were limited to short time bisection task: *WR *for short intervals was significantly lower for young controls (0.13 ± 0.02 ms) than for both elderly subjects (0.23 ± 0.02, p = 0.004) and AD patients (0.29 ± 0.04, p = 0.00006). A trend toward significance (*p = 0.05*) was also noted in the *WR *difference between the latter two groups. Finally, in contrast to young controls, showing comparable *WR*s for short (0.13 ± 0.02 ms) and long (0.10 ± 0.01 ms) time bisection tasks (*p = 0.5*), all other participants exhibited substantially different *WRs *in the two time bisection tasks. Specifically, both elderly subjects and AD patients displayed significantly higher *WR*s for short interval estimates (0.23 ± 0.02 ms and 0.29 ± 0.04 ms, respectively) than for long interval estimates (elderly controls: 0.13 ± 0.01 ms, p = 0.006; AD patients: 0.14 ± 0.03 ms, p = 0.0005). Therefore, whereas Weber's Law held for discrimination of both sub- and supra-second durations in young subjects, with variability in time representation increasing proportionally with the mean of the interval being represented, timing behavior of elderly subjects and AD patients, as studied by the present tasks, did not show any scalar property.

### Precision

The responses of AD patients were overall significantly less consistent (90.4 ± 2.2%) than responses given by elderly (95 ± 0.9%, p = 0.04) and young subjects (98 ± 0.6%, p = 0.002), who adopted almost the same decisional criterion over successive time discrimination trials [*GROUP main effect*: *F(2, 31) = 6, p = 0.006*].

None of the average scores obtained by AD patients in the neuropsychological test battery and in the scales for dementia assessment significantly correlated with any of the significant parameters (*WR and Precision*) of the time bisection tasks (*Pearson r *correlations, assessed at 5% alpha level).

## Discussion

The use of the time bisection procedure allowed for a detailed analysis of AD patients' timing behavior within the theoretical framework of Scalar Timing Theory, with particular attention devoted to the function of the memory components.

The results presented above indicate that patients diagnosed with probable mild Alzheimer's disease had diminished ability to discriminate intervals in the sub-second range, as shown by the substantially increased *WR *index in the short time bisection task, with respect to the long time bisection task. The same pattern of results was obtained for elderly participants. Thus, for these two groups, interval timing on different anchor durations was not properly scalar, that is, *DL *and *BP *did not co-vary proportionally in the sub- and supra-second time bisection tasks, with AD patients displaying the poorest sensitivity to ms-scale intervals, compared to both young and elderly controls. Furthermore, contrary to the other groups, AD patients generally failed to maintain a constant timing criterion over the same bisected duration, as indicated by the substantial imprecision in making both repeated short and long temporal judgments. In contrast, they did not systematically over- or under-estimate durations relative to control participants (*BP *for the three groups was comparable in both tasks).

Millisecond and second durations were used in our study to better isolate deficits specific to temporal processing from more generalized cognitive impairments. If timing difficulties of AD patients were mainly due to higher order cognitive impairments, their temporal performance should have been much worse for discrimination of supra-second intervals, that are known to require additional mnemonic and attentional demands to be correctly encoded. The obtained results suggest that this was not the case. Indeed, apart from an overall decreased precision of performance, that may be indicative of an unspecific deficit in sustaining attention throughout both short and long time bisection tasks, AD patients seemed to display more difficulties in timing millisecond durations. To a lesser extent, the same observation holds for the elderly group. Therefore, performance of AD patients and elderly subjects can be interpreted as primarily dependent on mechanisms specifically serving timing operations.

One of the advantages of the SET model is that it is modular, thus allowing to make assumptions about the correlation existing between a certain temporal behavioral pattern and the selective involvement of cognitive stages included in the model.

Studies investigating the effect of selective brain lesions or pharmacological manipulations on time bisection or peak-interval responses in animals have identified characteristic patterns of accuracy (under- or over-estimation) and variability (decreased or increased) of timing behavior that are specifically associated to clock [5,7,8] or memory [5,9-11] disruptions. It is assumed that clock variability is relatively small compared to memory variability, so that changes in clock speed mostly affect accuracy, horizontally displacing psychometric functions. This contrasts with the memory pattern which is represented by both accuracy and variability changes, that respectively modify horizontal position and slope (or shape) of temporal response curves. Furthermore, after the introduction of a variable that varies clock pulse discharge, accuracy is immediately altered, but it then returns to the criterion time as clock can be recalibrated quickly. On the contrary, changes in accuracy and variability induced by variables distorting pulse encoding into memory are gradual and long lasting, as memory-storage speed cannot be rapidly readjusted [26,27,45]. It has been additionally suggested that, within the memory pattern, time overestimation denoted by rightward shifts of the psychometric function, might depend on a selective reference memory breakdown following frontal lesions, whereas time underestimation and variability increase, that are mostly subsequent to medio-temporal lesions, might be interpreted as working memory deficits.

It is important to point out that a cautious approach should be adopted when comparing results obtained from animal and human studies. The effects of pharmacological manipulations in animals are generally studied under "state-change" design, where differences between conditions are amplified by the procedure of directly contrasting the behavioral effects produced on the same subject by two different drugs. In contrast, normal or pathological conditions in humans can be described as "steady-state" designs, where the observed behavioral effects are not experimentally produced. Thus, when employing time bisection with human patients, some of the above-mentioned SET predictions about the expected timing behavior should be partly reformulated. In particular, changes in the clock rate should not be evident from patients' bisection accuracy, since bisection point (denoting accuracy) is typically measured after that any hypothetical rescaling of distorted clock speed has occurred. Moreover, as already observed elsewhere [23], contrary to other tasks requiring a perceptual estimation of an objective duration, in which differences in clock speed are directly reflected by performance differences (e.g., a faster internal clock would correspond to overestimation of the to-be-timed interval), bisection judgments result from a comparison between two intervals (the reference and the test intervals) that have both been encoded according to subjective internal clock speed. Hence, independently of whether clock rate is distorted or not, the location of bisection point should not change significantly. In contrast, if memory or attention processes are influencing temporal performance, psychometric functions are expected to shift. For instance, working and reference memory disruptions may respectively alter the storage of the test and anchor durations, so that bisection responses will be given according to two different subjective time scales, consequently affecting bisection point. Finally, though conceptually very elegant, behavioral patterns predicted by SET through pharmacological manipulations are not so evident when analyzing patients' timing behavior and in practice it is difficult to separate clock from memory variance.

Given these observations, the lack of any significant shift in the bisection function of AD patients in the present study might be referred to different alternative interpretations.

First, a general functional drop extending to both the working and reference memory components due to a severe widespread decreasing cholinergic activity of the BFCS might have been responsible for compensating opposite horizontal shifts in the psychometric curves. In addition, although the patients included in the present study were all diagnosed with the same severity level of AD, the full extent of the pathology cannot be established with certainty in any subject and the degeneration might have differentially involved the regions of the BFCS, leading to the cancellation of specific temporal distortion when analysing the average behavioral performance. Alternatively, a very mild cholinergic dysfunction might have not been sufficiently important to produce any appreciable temporal distortion or to be detected by the time bisection tasks used here.

An impairment of the clock component is also possible, although the decreased temporal sensitivity of AD patients, especially observed in the domain of millisecond intervals, represents a hallmark of memory variance. Increased *WR *index and consequent violation of the scalar property in the short time bisection task might be indeed selectively due to a reference memory dysfunction, characterized by a greater degree of noise in the memory distributions of millisecond anchor durations (100 and 600 ms), compared to second anchor durations (1 and 3 s). Impaired temporal sensitivity displayed by a group of frontal lobe patients tested with both sub- and supra-second time bisection tasks has been interpreted as the effect of a potential damage at the reference memory system [24]. Further support to this interpretation is given by a number of evidences indicating that the frontal lobes, besides their very well known contribution to working memory, may be particularly crucial to form and manipulate temporal representations in reference memory [8,18,19,41].

On the other hand, a random error at the working memory level might be hypothesized, with AD patients failing to encode the correct number of pulses at the end of each presented millisecond duration, thus significantly increasing timing variability. Patients with resection of right medial-temporal lobe, a brain structure that is strongly implicated in working memory processes, were found to selectively increase *DL *and *WR *values when discriminating intervals within a duration range less than 1000 ms [20]. Additional evidence for an involvement of temporal lobe structures in the current behavioral pattern of results comes also from literature attempting to isolate neural systems that are used for timing durations at the sub-second and several second ranges. By means of fMRI, significantly greater activity has been observed in middle and superior temporal gyri during estimation of a 600-ms interval compared to a 3-s interval [21].

Finally, also a change in the decisional bias at the comparator level during the tasks, that might be related to AD patients' attentional disturbances, would have possibly both flattened bisection curves, as indexed by increased *WR *values, and increased the proportion of variance unexplained by the logistic model, as evidenced by decreased precision.

Our results are consistent with previous studies [34,36,37] showing that AD participants were more variable and imprecise in their time estimations than controls. Moreover, the present findings support the observation that AD can be associated with both under- and over-estimation or with no bias, but always in the framework of variability. For example, Carrasco and coworkers [34] found that some AD patients overproduced time, whereas others underproduced time, so that, on average, the AD group was not significantly less accurate in timing than the control group.

There is some evidence that timing also changes with age. Some authors have suggested that age-related changes in duration judgments depend on slowing down of the internal clock [46-49], some others have indicated that impaired temporal information processing in older adults may be mainly related to reduced attentional resources [50,51] or memory deficits [52,53]. Since in the present study the behavioral pattern of elderly participants (decreased sensitivity to millisecond-scale durations, with respect to young controls) is comparable, though milder, to that of AD patients, we put forward the same alternative hypotheses described above for explaining AD patients' performance.

Finally, it is important to note that the present results are not consistent with some previous findings showing that AD patients are compromised in judging durations in the range of seconds [34,36,37]. However, the majority of timing studies in AD have only employed durations in the range of several seconds, without no comparison with millisecond timing performance. Thus, as mentioned previously, it is possible that the timing deficits reported by those studies strongly depended on the additional cognitive resources required to encode longer durations, making difficult to isolate temporal perception dysfunctions from more generalized cognitive impairments.

## Limitations

It should be stressed that the absence of group differences in the location of bisection point, that would have been strongly indicative of the specific source of memory variance, may not only depend on the different experimental design employed in the present research with respect to animal studies. It also much relies on the level of degeneration that may differentially affect the brain cholinergic structures, as a function of the severity of AD and of the specific case history of single patients. In view of this, we suggest that future experiments aiming at testing SET with AD patients adopt a component analysis method as an alternative approach, wherein memory, attention and strategic processes are studied by means of other tasks and then correlated with timing measures obtained with different SET behavioral paradigms. In particular, both bisection and peak-interval procedures should be employed, as they may provide relevant complementary timing information.

## Conclusion

With this study we demonstrated that a group of patients with mild AD had decreased sensitivity in discriminating durations on a millisecond time scale, whereas they performed well in timing supra-second intervals. The primary aim of our work was to delineate the relative contribution of the working and reference memory components in time perception. However, we conclude that the timing pattern observed in AD participants could be well explained by functional changes taking place at different levels of the SET model: both clock, memory and decisional mechanisms might be impaired in AD.

As a final observation, the similarity between the timing patterns of elderly and AD participants raises the important issue of whether normal aging and AD are two distinct processes or differ only in the level of severity, forming one continuum. The present results seem to support the continuum hypothesis, at least in the domain of temporal information processing, suggesting that AD may be considered as part of the normal aging process.

## Competing interests

The authors declare that they have no competing interests.

## Authors' contributions

LC and LI wrote the manuscript, acquired, analysed and interpreted data. PN participated in the design of the experiment, helped interpreting data and revising the manuscript. All authors read and approved the final manuscript.
